# Diagnosing Bacterial Infection in the era of Pandemic: A Case Report

**DOI:** 10.7759/cureus.22567

**Published:** 2022-02-24

**Authors:** Junki Mizumoto, Hirohisa Fujikawa

**Affiliations:** 1 Department of Medical Education Studies, International Research Center for Medical Education, Graduate School of Medicine, The University of Tokyo, Tokyo, JPN; 2 Department of Internal Medicine, Suwa Central Hospital, Nagano, JPN

**Keywords:** gut feeling, sepsis, infectious diseases, diagnostic reasoning, covid blindness

## Abstract

In the coronavirus disease 2019 (COVID-19) pandemic era, physicians’ clinical decision-making is often distorted. A man in his 60s presented with an already-subsided cough and anxiety about COVID-19. The physician was influenced by the patient’s anxiety and stuck to exclusion of COVID-19. The patient was finally diagnosed with sepsis caused by obstructive pyelonephritis. The key point for diagnosis was physicians’ awareness that the patient took slow and heavy steps. To confront the challenge of making an appropriate diagnosis of bacterial infection in the era of COVID-19, physicians should be aware of diagnostic biases and watch patients’ general appearance closely.

## Introduction

In the coronavirus disease 2019 (COVID-19) pandemic era, physicians are forced to consider “to be COVID-19, or not to be COVID-19” in seeing patients with fever or other respiratory symptoms. We cannot overemphasize the importance of a timely and accurate diagnosis of COVID-19 [1]. Patients with COVID-19 often present nonspecific and untypical symptoms, and some patients do not have any symptoms. The pandemic compromises the physical and psychological safety of physicians and health system capacity, and then distorts clinical decision-making [1]. In addition, the use of basic medical instruments, including sphygmomanometer, may be restricted because these instruments should be used exclusively for each individual patient [2]. Herein, we report a patient who complained about fear of COVID-19. The patient did not have any specific symptoms. The physician was influenced by the patient’s word and stuck to exclusion of COVID-19 more than necessary, which almost led to an oversight of bacterial infection.

## Case presentation

A 68-year-old Japanese man complained of mild cough from four days to one day before. Although cough subsided, the patient was worried about whether he developed COVID-19 and he might pass the virus to his mother, whom the patient provided at-home daily care solely. At that time, COVID-19 was mildly spread in the town. His past medical history included myocardial infarction, diabetes mellitus, hypertension, bladder cancer (postoperative), and non-cancerous tumor of the colon (postoperative). He regularly took bisoprolol 5 mg/d, azilsartan 20 mg/d, amlodipine 10 mg/d, rosuvastatin 5 mg/d, metformin 500 mg/d, and voglibose 0.6 mg/d. His usual blood pressure was about 140/80 mmHg, and his heart rate was about 50 beats per minute. Although the patient was afebrile, we first saw him on full personal protective equipment in the tent only for febrile patients, for fear that the patient might develop COVID-19. The tent was not equipped with a sphygmomanometer to prevent contact infection.

The patient did not appear toxic and did not complain of any symptoms in the tent. The body temperature was 36.5°C, heart rate 84 beats per minute, respiratory rate 20 per minute, and oxygen saturation 99% while breathing ambient air. Upon physical examination, a normal pharynx was seen. Lung and heart sounds were normal. There was no rash or arthritis. The abdomen was not tender. Costovertebral angle tenderness was negative.

**Figure 1:**
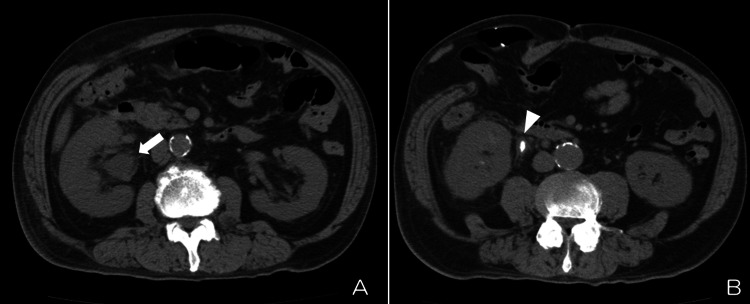
(A) Right hydronephrosis (white arrow). (B) Ureteral calculus causing hydronephrosis (white arrowhead)

Our hospital could not perform severe acute respiratory syndrome coronavirus 2 (SARS-CoV-2) real-time reverse transcription-polymerase chain reaction, and the SARS-CoV-2 antigen rapid test was negative. A chest X-ray was performed to rule out pneumonia, and no abnormal finding was seen. We were reassured by this negative result and thought that no additional evaluation might not be needed. We, however, became aware that the patient took slow and heavy steps to the X-ray room. On second thought, we paid attention to slight tachycardia (heart rate 84) regardless of his taking beta-blocker. We decided to reexamine the patient. It thus turned out that the blood pressure was 90/56 mmHg. The limbs were warm. Jugular vein distention was not seen. Considering slight tachycardia, blood pressure lower than usual, and the slow and heavy steps, we thought that the patient might fall into serious conditions such as distributive shock. The extracellular fluid infusion was started immediately, and blood laboratory tests were performed. The results were as follows: white blood cell counts of 25,240/µL (normal range: 3590-9640/µL), serum C-reactive protein level of 32.08 mg/dL (0-0.3 mg/dL), serum creatinine kinase level of 1443 IU/L (39-308 IU/L), serum lactate dehydrogenase level of 313 IU/L (85-227 IU/L), and serum creatinine level of 3.05 mg/dL (0.6-1.1 mg/dL). Lactic acid was not checked. A computed tomography scan revealed right hydronephrosis due to ureteral calculus of 5 mm in diameter (Figure 1). Urinalysis revealed pyuria and bacteriuria. The diagnosis of obstructive pyelonephritis, sepsis, and distributive shock was made.

An intravenous antibiotic (tazobactam/piperacillin) was administered by urologists, and a percutaneous nephrostomy was performed. A blood culture turned out to be positive in *Escherichia coli*. The patient was recovered in a week and was discharged. After discharge, the patient did not develop fever, malaise, or other symptoms again.

## Discussion

This case illustrated a man with an already-subsided cough and anxiety about COVID-19 who was finally diagnosed as a distributive shock due to bacteremia caused by obstructive pyelonephritis. Not measuring blood pressure routinely for fear of COVID-19 contagious infection might delay a correct and prompt diagnosis. The key point for diagnosis was the patient’s slow and heavy steps. It is probably because the bacteremia increased the patient’s demand for oxygen, although the patient did not complain of general malaise, difficulty in moving, or dyspnea on exertion. Closely watching patients’ general appearance may be an essential diagnostic strategy. Physicians’ gut feeling is one composite of complicated processes in diagnosing sepsis [3]. Physicians should be aware that less direct interactions between patients and medical staff may lead to missed or delayed diagnoses.

Tachycardia due to distributive shock was obscured by his regular medication. Patients taking beta-blockers may not present tachycardia appropriate for falling blood pressure [4]. Although mild tachycardia is a nonspecific finding, the gap between the patient’s heart rate daily and at the time of presentation had an important implication.

In the pandemic era, physicians may fall into “COVID blindness” [5], or being satisfied with the diagnostic label of “COVID-19 rule-out.” It is reported that this diagnostic bias leads to delayed treatment of bacteremia [6]. In this case, an appropriate evaluation was postponed until the result of the antigen rapid test was turned out, although the probability of COVID-19 was low. We decided to see the patient in the tent with few equipment only because the patient complained of the fear of COVID-19. That is, we overreacted to the word “COVID-19” that the patient said. However, we finally reached the right diagnosis. Physicians should not complete the diagnostic process by confirming a negative chest X-ray or virological tests and should consider other alternative diagnoses to avoid COVID blindness [1,7].

The patient visited our hospital regardless of subsided symptoms and his mother in need of care. When a patient with a high threshold for seeking medical care visits a medical institution for a minor symptom, the patient may not be able to verbalize his or her symptoms but have a dangerous disease, like this case. This new method of clinical reasoning based on patients’ care-seeking behavior is called behavior-based medical diagnosis [8]. In this case, the behavior-based medical diagnosis might have enabled us to start treatment for sepsis earlier on. In hindsight, the patient might feel general fatigue or discomfort that could not be verbalized. We guess that the patient subconsciously invented the reason for anxiety about COVID-19 to convince us why he was seeking medical care.

In the pandemic era, febrile patients often face some hurdles of medical care access. The lack of medical resources to see febrile patients and patients’ refrainment from going out due to fever may lead to diagnostic delay and poorer outcomes [9-11]. The coexistence of COVID-19 and bacterial infection may be another factor of poorer outcomes [12]. The coexistence develops two potential problems: a bacterial infection masks COVID-19 and delays isolation of the patient; COVID-19 masks a bacterial infection and delays appropriate therapy.

## Conclusions

Physicians confront the challenge of making a correct and prompt diagnosis of bacterial infection in the era of COVID-19. Tachycardia due to septic shock may be obscured by regular intake of beta-blockers. Avoiding COVID blindness and enhancing the sensitivity of gut feeling by closely watching patients’ general appearance may be needed.
